# The Potential Regulatory Mechanism of lncRNA 122K13.12 and lncRNA 326C3.7 in Ankylosing Spondylitis

**DOI:** 10.3389/fmolb.2021.745441

**Published:** 2021-10-21

**Authors:** Jian-xiong Wang, Feng-yang Jing, Yue-chen Xu, He-xiang Zong, Yi-ran Chu, Cong Wang, Ke-ming Chen, Wan-qiu Tong, Xi-le Wang, Sheng-qian Xu

**Affiliations:** ^1^ Department of Rheumatology and Immunology, The First Affiliated Hospital of Anhui Medical University, Hefei, China; ^2^ Department of Dental Implant Center, Key Laboratory of Oral Diseases Research of Anhui Province, Stomatologic Hospital and College, Anhui Medical University, Hefei, China; ^3^ Department of Radiation Oncology, The First Affiliated Hospital of Anhui Medical University, Hefei, China

**Keywords:** ankylosing spondylitis, long noncoding RNA, RNA sequencing, ceRNA signal network, nomogram

## Abstract

This work aims to analyze and construct a novel competing endogenous RNA (ceRNA) network in ankylosing spondylitis (AS) with bone bridge formation, lncRNA. Using RNA sequencing and bioinformatics, we analyzed expression profiles of long noncoding RNAs (lncRNAs), microRNAs (miRNAs), and mRNAs in whole blood cells from 5 AS patients and 3 healthy individuals. Next, we verified the expression levels of candidate lncRNAs in 97 samples using the ΔΔCt value of real-time quantitative polymerase chain reaction (qRT-PCR). We used multivariate logistic regression analysis to screen lncRNAs and clinical indicators for use in the prediction model. Both SPSS 24.0 and R software were used for data analysis and prediction model construction. The results showed that compared with the normal controls, 205 long noncoding RNAs (lncRNAs), 961 microRNAs (miRNAs), and 200 mRNAs (DEmRNAs) were differentially expressed in the AS patients. We identified lncRNA 122K13.12 and lncRNA 326C3.7 among 205 lncRNAs differentially expressed between AS patients and healthy humans. Then, we noted that 30 miRNAs and five mRNAs formed a ceRNA network together with these two lncRNAs. These ceRNA networks might regulate the tumor necrosis factor (TNF) signaling pathway in AS development. In addition, the expression level of lncRNA 122K13.12 and lncRNA 326C3.7 correlated with various structural damage indicators in AS. Specifically, the lncRNA 326C3.7 expression level was an independent risk factor in bone bridge formation [area under the ROC curve (AUC) = 0.739 (0.609–0.870) and *p* = 0.003], and the best Youden Index was 0.405 (sensitivity = 0.800 and specificity = 0.605). Moreover, we constructed a lncRNA-based nomogram that could effectively predict bone bridge formation [AUC = 0.870 (0.780–0.959) and *p* < 0.001, and the best Youden Index was 0.637 (sensitivity = 0.900 and specificity = 0.737)]. In conclusion, we uncovered a unique ceRNA signaling network in AS with bone bridge formation and identified novel biomarkers and prediction models with the potential for clinical applications.

## Introduction

Ankylosing spondylitis (AS) is one of the primary subtypes of spondyloarthritis (SpA), and its diagnosis is still based on the modified New York 1984 criteria (Linden et al., 1984). Therapeutic decisions in newly diagnosed AS are based upon whether there is active disease (believed to be closely related to disease prognosis). However, in clinical practice, even in some patients with controlled disease activity, radiographic characteristics in the sacroiliac joints and syndesmophytes gradually appeared, which eventually developed into a bone bridge (Poddubnyy et al., 2011). Therefore, prior identification of patients who may form bone bridges is critical for clinical practice. According to some AS-related studies, the genes responding to the environmental prompts (such as long-term mechanical stress or the long course of the disease) might affect epigenetic modifications at the bone–cartilage interface (Sieper and Poddubnyy, 2017). The key epigenetic regulators have included long noncoding RNAs (lncRNAs) that have been shown to have multiple biological functions (Yu et al., 2018; Liu et al., 2021). Some important lncRNAs have been observed in AS, and these might be involved in the pathogenesis of AS. These genetic biomarkers of AS pathogenesis include programmed cell death 1 (PDCD1), DNA methyltransferase 1 (DNMT1), caspase recruitment domain-containing protein 11 (CARD11), and phospholipase Cγ1 (PLCG1) (Xu et al., 2019). Additionally, several of the lncRNAs also focused on inflammation-related disease activity. One study showed that the overexpression of the lncRNA NKILA in AS was associated with active disease and predicted the duration of treatment (Gai and Li, 2019). However, new bone formation was not only produced via inflammatory-dependent pathways (Poddubnyy and Sieper, 2017). This is because it is still difficult to detect bone bridges in advance at the early stages because of the lack of accurate and predictable biomarkers. The currently insufficient prediagnosis that could prevent bone bridge formation and the close relationship between lncRNAs and AS support the requirement for further understanding of lncRNA. Some previous studies have illustrated that lncRNAs were engaged in ceRNA networks in many diseases (Zhao et al., 2021). These lncRNAs might act as competing endogenous RNAs (ceRNAs) by competitively binding to miRNAs through their miRNA response elements, thus regulating the expression levels of miRNA-target mRNAs (Ju et al., 2020). There were fewer reports of ceRNA networks in arthritis, and these mainly focused on osteoarthritis and rheumatoid arthritis. As the ceRNA network plays an important role in other rheumatoid arthritis, many essential lncRNAs have also been found in AS. Therefore, the lncRNA–miRNA–mRNA interaction may be an important mechanism underlying the initiation and development of AS. However, the status quo that the ceRNA network is associated with bone bridge formation has not been investigated.

Consequently, this work attempted to find a reliable prognostic biomarker using RNA sequencing (RNA-seq) and identify the lncRNA–miRNA–mRNA interaction mechanism in AS. Through clinical validation, we developed an lncRNA-based nomogram to predict bone bridge formation.

## Materials and Methods

### Patient Recruitment and the Ethics Statement

This case-control study included 32 healthy humans and 73 patients. All healthy humans were recruited from the physical examination center of the First Affiliated Hospital of Anhui Medical University from October 2020 to January 2021. None of the patients suffered from acute or chronic diseases or took oral medication. All AS patients met the modified New York 1984 criteria (Linden et al., 1984) [①Diagnosis. 1) Clinical criteria: a) low back pain and stiffness for more than 3 months, which improves with exercise but is not relieved by rest. b) Limitation of motion of the lumbar spine in both the sagittal and frontal planes. c) Limitation of chest expansion relative to typical values corrected for age and sex. 2) Radiologic criterion: sacroiliitis grade ≥2 bilaterally or sacroiliitis grade 3–4 unilaterally. ②Grading. 1) Definite ankylosing spondylitis if the radiologic criterion is associated with at least 1) clinical criterion. 2) Probable ankylosing spondylitis if a) three clinical criteria are present. b) The radiologic criterion is present without any signs or symptoms satisfying the clinical criteria (other causes of sacroiliitis should be considered.)]. The patients were distinguished by examining the presence of bone bridges using X-ray (pelvis and whole spine), including 30 AS patients with bone bridge formation and 43 patients without bone bridge formation, who were also recruited from the Department of Rheumatology and Immunology of the First Affiliated Hospital. Clinical records were collected, and the subjects were interviewed by professional rheumatologists using questionnaires. All the patients were first examined, and they were excluded from the study if they had infectious diseases, serious kidney diseases, serious liver diseases, or endocrine disorders. Moreover, if important data were missing or patients were treated with steroids, nonsteroidal anti-inflammatory drugs (NSAIDs), or disease-modifying antirheumatic drugs (DMARDs) within a specific period, they were also eliminated. In this study, each participant provided informed consent to participate, and the study was performed in accordance with the Declaration of Helsinki. The study protocol was approved by the Ethics Committee of Anhui Medical University (No. 20200740).

### RNA Extraction, Library Construction, and Sequencing

In the screening phase, whole peripheral blood cells from three healthy humans and 5 AS patients were used for RNA-seq. Total RNA was treated with RQ1 DNase (Promega) to remove DNA. The quality and quantity of the purified RNA were determined by measuring the absorbance at 260 nm/280 nm (A260/A280) using a Smartspec Plus (BioRad). RNA integrity was further verified using 1.5% agarose gel electrophoresis. For each sample, 1 μg of total RNA was used for RNA-seq library preparation. Ribosomal RNAs were depleted with a Ribo-off™ rRNA depletion kit (Vazyme, N406-01). The purified RNA was used for the directional RNA-seq library preparation using a KAPA Stranded mRNA-Seq Kit for Illumina® Platforms (KK8544). Polyadenylated mRNAs were then purified and fragmented. Fragmented mRNAs were converted into double-stranded cDNA. Following end repair and A tailing, the DNAs were ligated to a Diluted Roche Adaptor (KK8726). After purification of the ligation product and size fractioning to 300–500 bps, the ligated products were amplified, purified, quantified, and stored at −80°C before sequencing. The strand marked with dUTP (the second cDNA strand) was not amplified, allowing strand-specific sequencing. For high-throughput sequencing, the libraries were prepared following the manufacturer’s instructions and applied to an Illumina Novaseq 6000 system for 150 nt paired-end sequencing.

### Identification of Differentially Expressed Candidate lncRNAs

We searched for coding genes 10-kb upstream and downstream of all the identified differentially expressed lncRNAs (DElncRNA). The Pearson correlation coefficient between DElncRNA and mRNA was calculated for coexpression analysis. The pair of lncRNA–target relationships that had a correlation coefficient > 0.6 and *p* < 0.05 was selected, and then, the union of the two data sets was taken to obtain the cis-acting target of lncRNA. The predicted DElncRNA and coexpressed genes formed gene lists and were input into the Kyoto Encyclopedia of Genes and Genomes (KEGG, www.kegg.jp) for KEGG pathway analysis and Gene Ontology (GO, geneontology.org) for GO analysis. The KEGG pathways and GO terms with *p* < 0.05 were considered to be significantly enriched. The final output data were used to select the candidate lncRNAs.

### miRNA Prediction and ceRNA Network Construction

We searched for the most lncRNA-related miRNA by using DIANA-miRPath v2.0, which provided for the first time a series of precise tools for miRNA-targeted pathway analysis via a web interface and could be accessed at www.microrna.gr/LncBase (Paraskevopoulou et al., 2016). A ceRNA network was then constructed using Cytoscape software (version 3.6.1) to explore the functions of the differentially expressed genes.

### Validation of Candidate lncRNAs by qRT-PCR

In the clinical phase, whole peripheral blood cells from 68 AS patients and 29 healthy humans were used. The total RNA in peripheral blood mononuclear cells from 97 samples was immediately extracted using the TRIzol method (Carlsbad, CA, United States) for qRT-PCR. RNA purity was checked by measuring the absorbance at A260/A280 using a Thermo Fisher Scientific NanoDrop One (Madison, WI 53711 Assembled in the United States). Total RNA was reverse-transcribed into cDNA using a PrimeScript™ RT Master Mix (TAKARA, Japan). qRT-PCR was performed using a TB Green^®^ Premix Ex Taq™ II(TAKARA, Japan). Last, the relative gene expression levels were calculated using the 2^−ΔΔCt^ method (ΔCt = Ct_target_ − Ct_reference_, −ΔΔCt = sample ΔCT − β-actin ΔCT) (Schmittgen and Livak, 2008), and the primer information is shown in Supplementary Table S5.

### Identification and Assessment of the Candidate DElncRNA

SPSS (version 24.0) was utilized for statistical analysis. The sociodemographic and clinical variables were computed using descriptive statistics (continuous and categorical variables were expressed as the median and frequency (%) and interquartile range P_75_−P_25_, respectively). A nomogram was adapted to detect the gender, the age of symptom onset, the age of definite diagnosis, the course of the disease, the lncRNA relative expression value, the human leukocyte antigen-B27 (HLA-B27) state, the visual analog score (VAS) for low back pain, ESR (mm/h), CRP (mg/L), and the diagnostic delay (defined as the time between the age of AS definite diagnosis and the onset of low back pain symptoms). The Ankylosing Spondylitis Disease Activity Score with the C-reactive protein (ASDAScrp) included patient‐reported assessments of CRP, duration of morning stiffness, back pain, general well‐being, and peripheral joint pain and/or swelling (Ramiro et al., 2014).

All patients received X-ray examinations for the pelvis and whole spine (from the cervical vertebra to lumbar vertebra, both posteroanterior and lateral). According to plain radiographs, the degree of sacroiliitis was categorized into five grades (0 = normal, 1 = suspicious, 2 = minimal, 3 = moderate, and 4 = ankylosis) (Sieper et al., 2009). Furthermore, the modified Stoke Ankylosing Spondylitis Spine Score (mSASSS) of the lumbar vertebra was used to evaluate the structural damage of AS (Creemers et al., 2005). Bone marrow edema or fat deposition in the sacroiliac joint was detected by magnetic resonance imaging (MRI) and scored according to the Spondyloarthritis Research Consortium of Canada (SPARCC) (Maksymowych et al., 2005).

T-tests or other nonparametric tests were used for distributed data (normal or skewed). Qualitative data were analyzed using the chi-square test. Linear regression was used to evaluate the value of lncRNA in reflecting disease activity (ASDAScrp) and structural damage, and logistic regression was used to evaluate the independent association with structural damage (mSASSS). The statistical significance was assumed to be *p* < 0.05 in all analyses. To further evaluate the predictive performance of the candidate DElncRNA plasma levels, we plotted bone bridge formation-dependent receiver operating characteristic (ROC) curves and calculated the area under the curve (AUC) for values in each dataset.

### Development of the lncRNA-Based Predictive Nomogram

We examined conventional clinical risk indicators and candidate DElncRNA values to determine independent predictors of bone bridge formation through univariate and multivariate logistic regression. A predictive nomogram was then established using R software. The nomogram was assessed with calibration curves, and we also measured the AUC value according to the ROC curve.

## Results

### Overview of RNA-Seq and Selection of Candidate lncRNA

**FIGURE 1:**
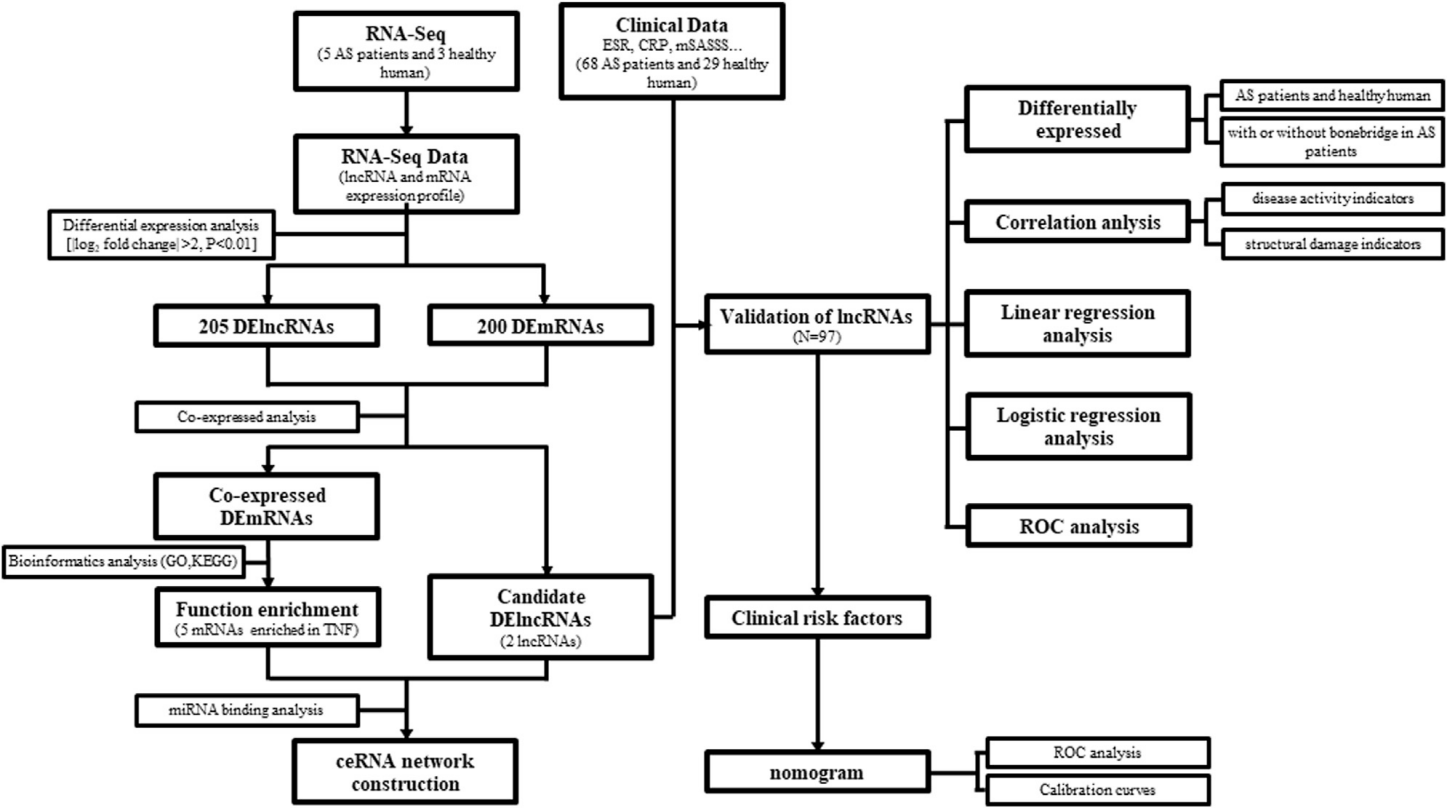
Overall design and flowchart.

**FIGURE 2:**
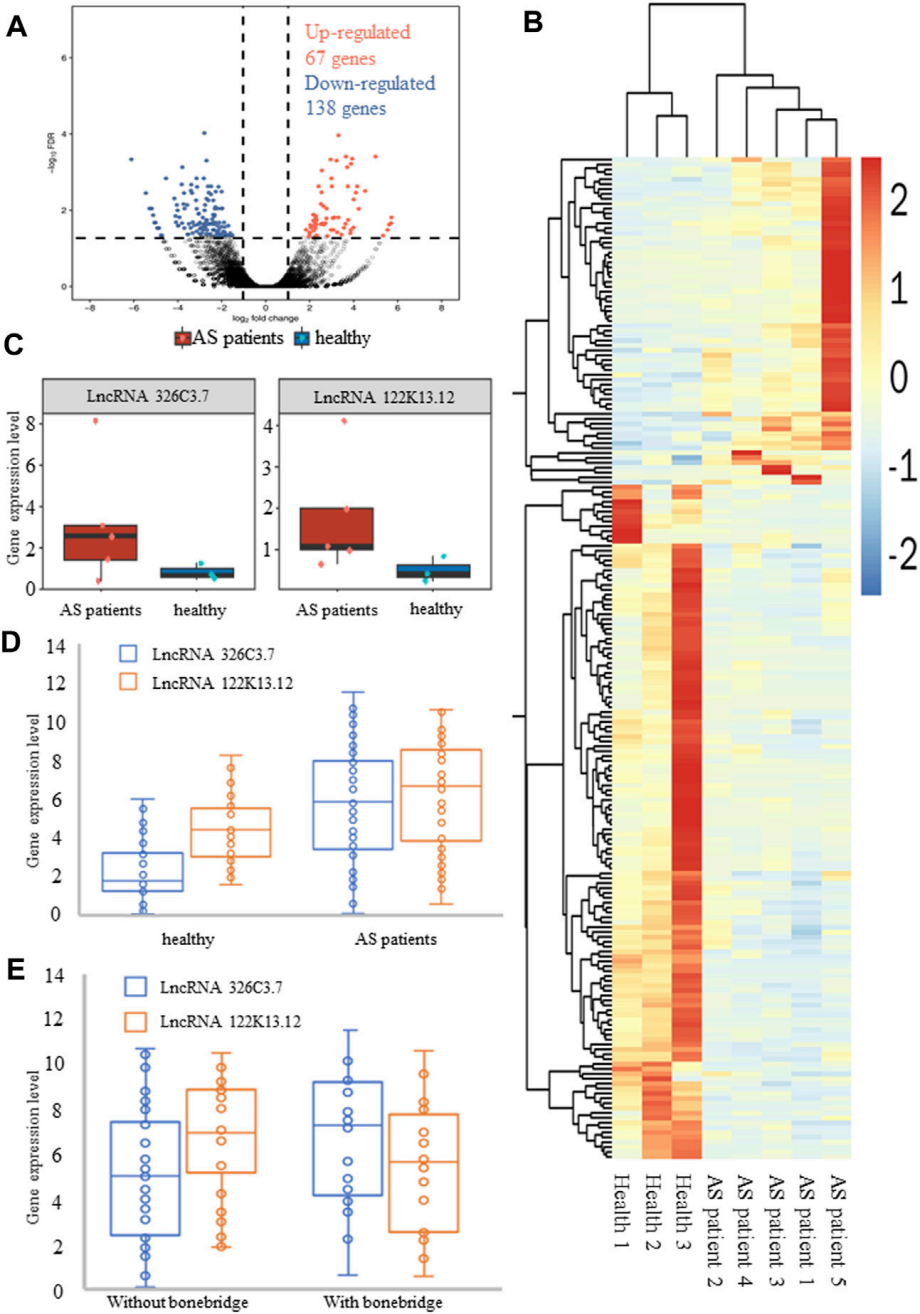
Differentially expressed genes in normal groups and patients with AS. **(A)** Volcano plot of differentially expressed lncRNAs. **(B)** Heatmap of differentially expressed lncRNAs. Blue and red indicate downregulated and upregulated lncRNAs, respectively. **(C)** RNA-sequencing data showed the expression levels of lncRNA 326C3.7 and lncRNA 122K13.12 in AS patients and healthy people. These two lncRNAs were overexpressed in AS patients. **(D, E)** Further verification of gene expression levels by qPCR. Also, two comparisons were made, one between healthy people and AS patients and the other between AS patients with and without bone bridge formation. The results, consistent with the results of sequencing, showed that these two lncRNAs were actually overexpressed in AS patients, particularly in patients with bone bridge formation.

The overall design and flowchart are presented in Figure 1. In total, we identified lncRNA and mRNA expression profiles from five AS patients and three healthy humans using RNA-seq. In total, 4694 novel lncRNAs were obtained, including 205 DElncRNAs with an adjusted p-value <0.01 and log_2_|fold change| > 2, and 200 differentially expressed mRNAs (DEmRNAs) were selected using the same method. Of the 205 DElncRNAs, the number of downregulated lncRNAs was higher than that of the upregulated lncRNAs, containing 67 upregulated lncRNAs and 138 downregulated lncRNAs. The DElncRNAs (volcano and heatmap plots) were visualized using the “ggplot2” and “pheatmap” packages of R software (Figures 2A,B).

Functional analysis showed that DElncRNA had 542 enriched GO terms, and coexpressed genes were enriched in 6733 GO terms that encompassed a variety of biological processes, including some of the essential terms such as response to a mechanical stimulus (GO:0009612), fat cell differentiation (GO:0045600), osteoblast differentiation (GO:0001649), and proliferation (GO:0033687). Additionally, DElncRNAs were enriched in 42 KEGG ID, and coexpressed genes were enriched in 279 ID, including the TNF pathway, IL-17 signaling pathway, osteoclast differentiation, Wnt pathway, and PPAR pathway (Supplementary Table S6). Combined with functional analysis results related to bone formation in AS pathology and consistent expression of lncRNA, we further selected two DElncRNAs that met the criteria to verify: ENSG00000254910 (lncRNA 326C3.7) and ENSG00000278238 (lncRNA 122K13.12). The expression of these lncRNAs in AS patients was significantly higher than that in healthy humans (Figure 2C).

### Identification and Validation of the Biological Function of DElncRNAs

The DElncRNA expression levels were examined by qPCR in 29 healthy humans and 68 AS patients, including 30 patients with syndesmophytes. The results confirmed that lncRNA 326C3.7 (5.798 vs. 2.211, *p* < 0.001) and lncRNA 122K13.12 (4.327 vs. 6.243, *p* = 0.001) were expressed and showed differential expression between patients and healthy humans (Figure 2D). Our results showed that the candidate DElncRNA was the same as the sequencing phase results and indicated that the identified DElncRNAs were expressed in vivo. Further comparison showed that lncRNA 326C3.7 was significantly different (*p* < 0.05) between AS patients with and without bone bridge formation (7.288 vs. 5.013 and *p* = 0.005, Figure 2E).

**FIGURE 3:**
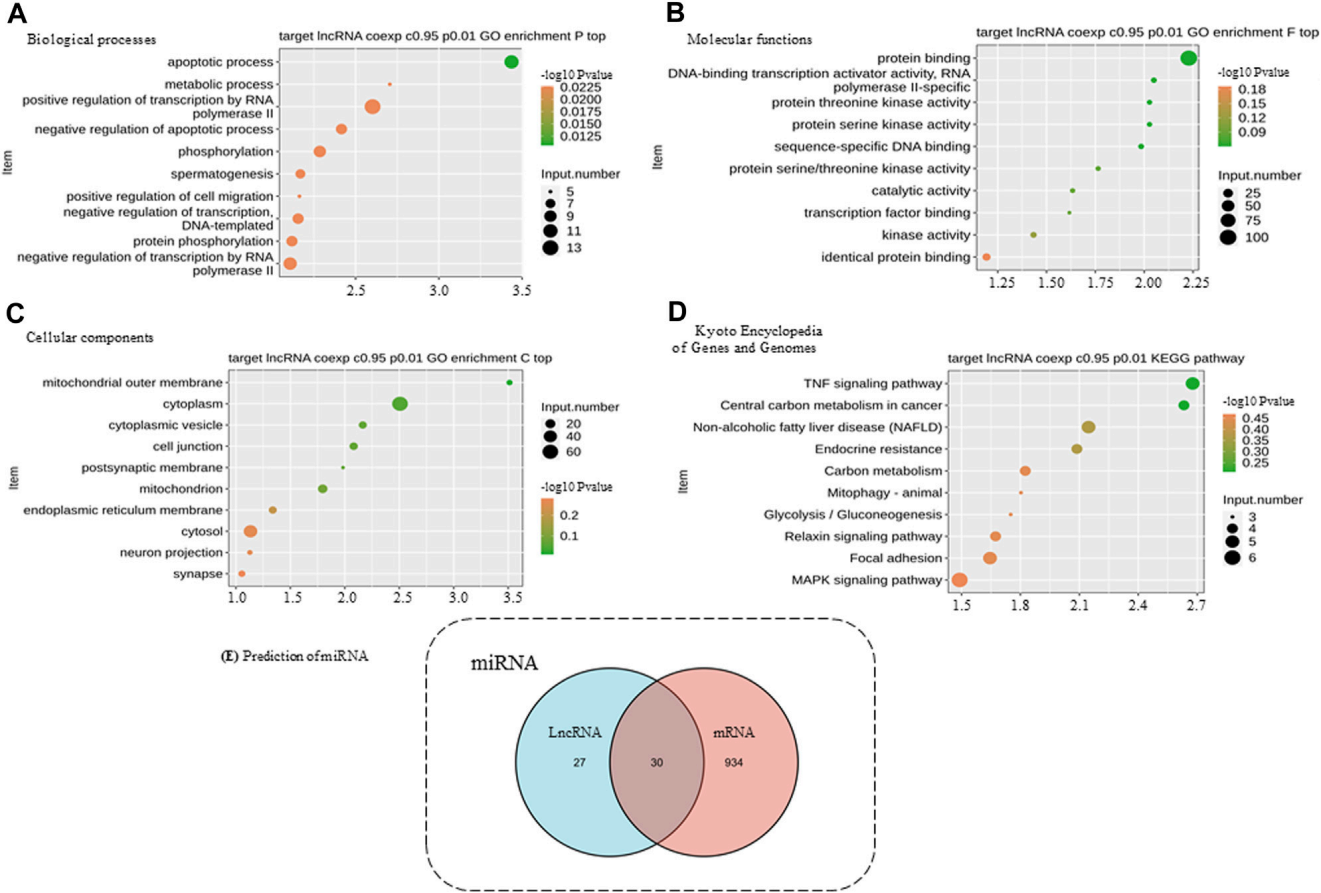
Functional enrichment analysis of differentially expressed genes and miRNA prediction. **(A)** BP, biological processes. **(B)** MF, molecular functions. **(C)** CC, cellular components. **(D)** KEGG, Kyoto Encyclopedia of Genes and Genomes. **(E)** DIANA databases were used to predict target miRNA. The numbers of predicted target miRNAs are presented in a Venn diagram. The predicted results of lncRNAs and mRNAs were integrated, and only the intersections were selected to increase the specificity.

The coexpression analysis of the two lncRNAs utilized the DEmRNA within the upstream and downstream 10-kb, and these were used for enrichment analysis (Figure 3). GO analysis showed that binding proteins (GO: 0005515) might be involved in disease through RNA-binding proteins. The results of KEGG analysis showed that the TNF signaling pathway (ID: hsa04668) was significantly enriched, which is a core signaling pathway involved in the systemic pathological process of AS. It is also enriched in the osteogenesis-related MAPK signaling pathway (ID: hsa04010).

### Exploration of the Potential Mechanism of ceRNA in AS

**FIGURE 4:**
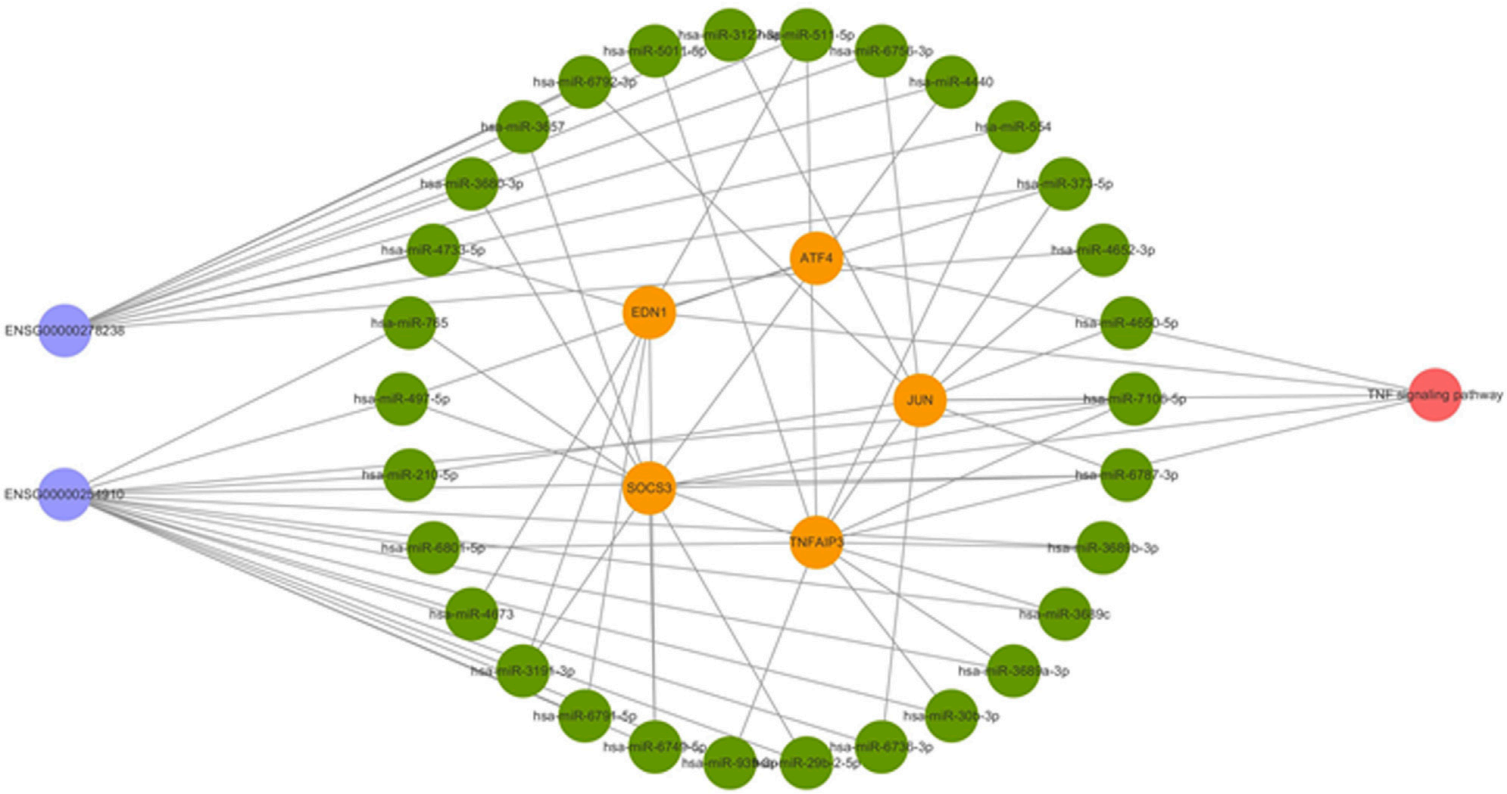
Competing endogenous RNA network in AS. The ceRNA network is based on lncRNA–miRNA–mRNA interactions. Blue represents lncRNAs, green represents miRNAs, yellow represents mRNAs, and red represents the signaling pathway.

We selected the five mRNAs significantly enriched in the TNF signaling pathway for further analysis together with lncRNAs. Using the bioinformatics website (http://carolina.imis.athena-innovation.gr/), the related miRNAs were predicted. A total of 30 miRNAs were predicted to be related to these five mRNAs and two lncRNAs (Figure 3E). Therefore, we speculated that lncRNA 326C3.7 and lncRNA 122K13.12 might be associated with mRNA through these miRNAs and may jointly participate in the pathogenesis of AS by regulating the TNF signaling pathway, which is constructed using the ceRNA signal network (Figure 4).

### Validation of the Clinical Value of Candidate lncRNAs

**FIGURE 5:**
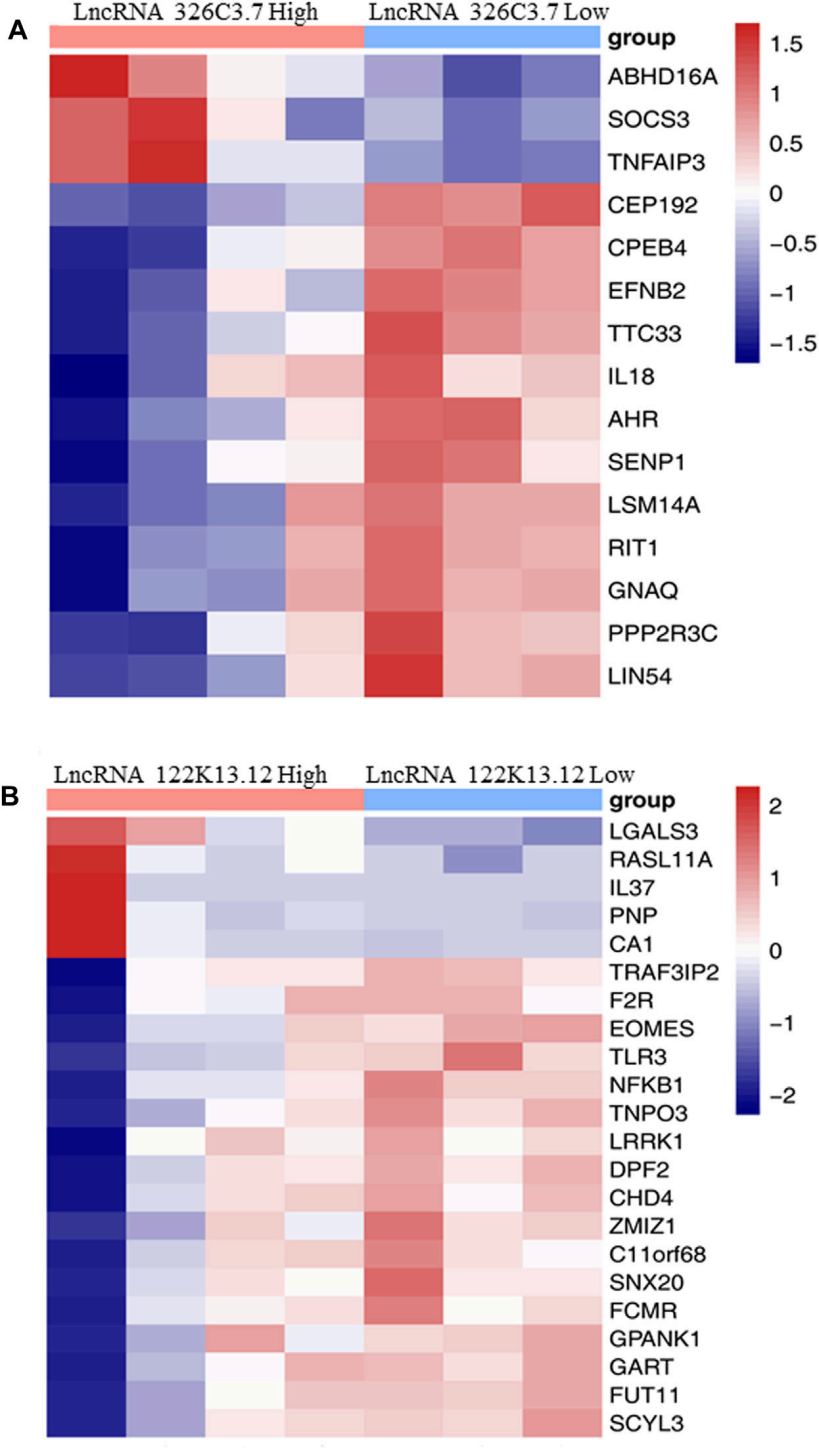
Heatmap showing the association between the expression level of target lncRNAs with AS-related biomarkers. **(A)** Heatmap showing the association between the expression level of lncRNA 326C3.7 with AS-related biomarkers. **(B)** Heatmap showing the association between the expression level of lncRNA 122K13.12 with AS-related biomarkers.

We analyzed the relationship between the expression of two lncRNAs and biomarkers in AS (Figure 5). The results showed that the expression levels of lncRNA 326C3.7 and lncRNA 122K13.12 were significantly correlated with many AS biomarkers. In particular, lncRNA 326C3.7 expression levels were correlated with ABHD16A, SOCS3, TNF AIP3, CEP192, CPRB4, EFNB2, TTC33, IL18, AHR, SENP1, LSM14A, RIT1, GNAQ, PPP2R3C, and LIN54. Among these genes, SOCS3 and IL18 were already viewed as the critical genes in AS. Next, we observed the candidate lncRNA expression levels and clinical data from AS patients. Some clinical characteristics differed significantly between AS patients with or without bone bridge formation, including lncRNA 326C3.7 expression, time of delayed diagnosis, mSASSS score, X-ray stage, and bone marrow edema in MRI (all *p* < 0.05). lncRNA 122K13.12 also showed partial differences among groups (*p =* 0.057). The detailed clinical characteristics are listed in Supplementary Table S1.

**FIGURE 6:**
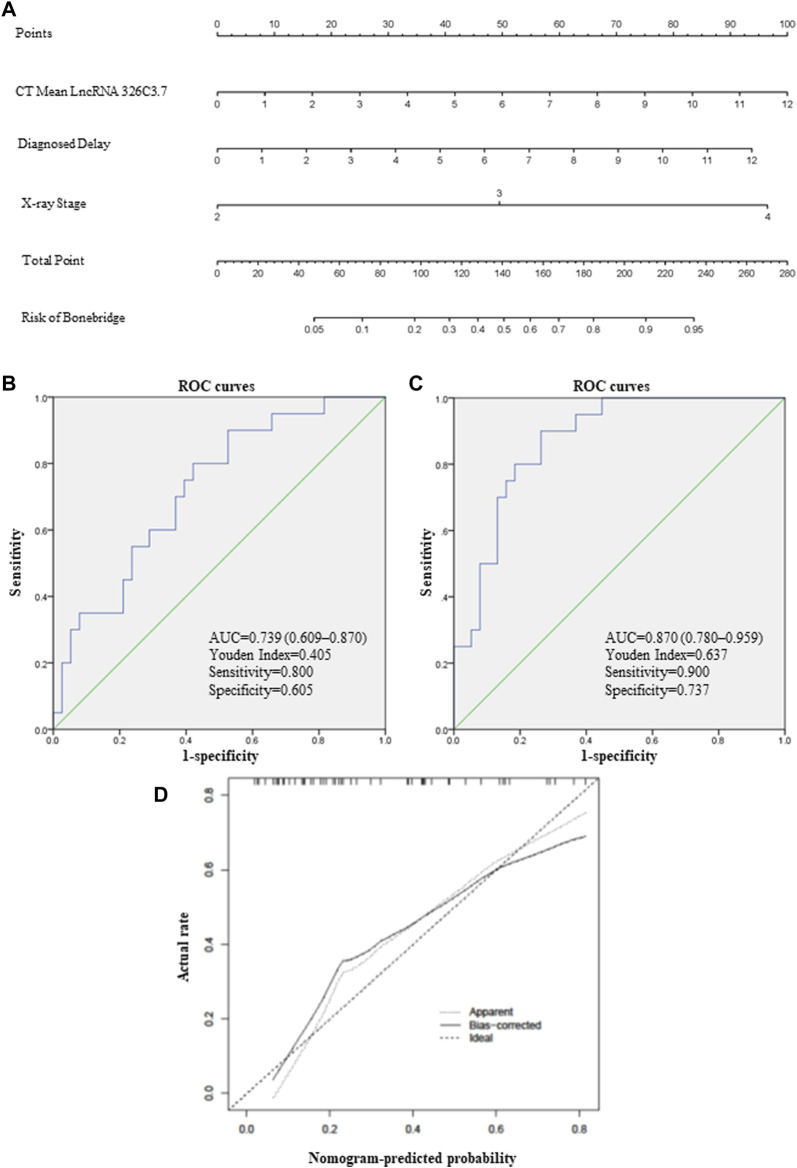
lncRNA-based nomogram to predict bone bridge formation in AS patients. **(A)** Nomogram, a visualization of the logistic regression model. Instructions: locate each characteristic on the corresponding variable axis, and draw a vertical line upward to the point’s axis to determine the specific point value. Repeat this process. Tally up the total point’s value, and locate it on the total point’s axis. **(B)** ROC curve depicting the predictive accuracy of lncRNA 326C3.7. AUC, area under the curve. **(C)** ROC curve depicting the predictive accuracy of the nomogram. **(D)** Calibration plot of the nomogram for predicting bone bridge formation. The 45-degree dotted line represents a perfect prediction, and the solid line represents the nomogram’s predictive performance.

A correlation analysis was used to determine the relationship between candidate lncRNAs and clinical indicators to identify the clinically related lncRNA. These two candidate lncRNAs were all correlated with some indicators of structural damage but not with indicators of disease activity (Supplementary Table S2). Specifically, lncRNA 326C3.7 was positively correlated with multiple variables, including X-Ray stage (*r* = 0.381 and *p* = 0.004), mSASSS (*r* = 0.390 and *p* = 0.003), SPARCC (*r* = 0.380 and *p* = 0.003), bone bridge formation (*r* = 0.380 and *p* = 0.003), VAS (*r* = 0.371 and *p* = 0.005), and time of delayed diagnosis (*r* = 0.371 and *p* = 0.005). Combined with differential expression results, we selected lncRNA 326C3.7 for the next step of linear and logistic regression. Defining the mSASSS score as the dependent variable to evaluate risk factors for the structural damage, we included lncRNA 326C3.7 and clinical indicators into the linear regression model (Supplementary Table S3). Our results with lncRNA 326C3.7 expression levels and ASDAScrp scores in the linear regression model showed a positive correlation (*R* ^*2*^ = 0.252, *F* = 7.430, and *p* = 0.002). Therefore, we speculated that the higher the expression of lncRNA 326C3.7, the more severe the degree of structural damage. Furthermore, we tested whether the prognostic performance of lncRNA 326C3.7 was independently correlated with bone bridge formation by univariate and multivariate logistic regression analyses. Considering the correlation of lncRNA, we selected the indicators according to correlation analysis. The results demonstrated that the HR of lncRNA 326C3.7 was 1.324 (*p* = 0.048, 95% CI = 1.003–1.748), and time of delayed diagnosis and X-ray stage were also significant risk factors because of the close correlation with bone bridge formation (*p <* 0.05). This indicated that lncRNA 326C3.7 could independently predict the prognoses of structural damage in AS (Supplementary Table S4). Last, we calculated the AUC and cut-off values to assess the specificities and sensitivities of gene predictive performance with bone bridge formation. Figure 6B shows that the AUC was 0.739 (95% CI = 0.609–0.870 and *p* = 0.003), and the best Youden Index was 0.405 (sensitivity = 0.800 and specificity = 0.605).

### Development of a Nomogram Combining lncRNA 326C3.7 With Clinical Risk Factors

Clinical risk factors such as the X-ray stage and delayed diagnosis time were vital predictors of bone bridge formation in AS patients. Therefore, we integrated these traditional risk factors with lncRNA 326C3.7 to develop a simple and efficient quantitative method for predicting bone bridge formation. Ultimately, based on gene and other logistic regression results, we developed an lncRNA 326C3.7-based nomogram (Figure 6A), which integrated lncRNA 326C3.7 and two clinical risk factors (X-ray stage and time of delayed diagnosis). The nomogram showed that the expression of lncRNA 326C3.7 was the most important, followed by the time of delayed diagnosis and X-ray stage closely. This user-friendly graphical tool allowed us to quickly determine the bone bridge formation probability for each AS patient. Moreover, we evaluated the calibration ability and the discrimination of nomograms using calibration plots and ROC curves. As shown on calibration plots, the probabilities determined by the nomogram were very close to the actual probabilities (Figure 6D). We further assessed the specificities and sensitivities of nomograms in the ROC curve analysis (Figure 6C). The AUC of the nomogram was 0.870 (95% CI = 0.780–0.959, *p* < 0.001), and the best Youden Index was 0.637 (sensitivity = 0.900 and specificity = 0.737).

## Discussion

Currently, AS is an incurable and debilitating autoimmune disease, and the treat-to-target approach relies on the ASDAS, which may reflect the disease activity (Ward et al., 2019). Higher disease activity led to more severe structural damage, but control of disease activity could not inhibit structural progress completely (Ramiro et al., 2014). A recent study found that new bone formation in AS might be through noninflammatory-dependent pathways (Linde et al., 2009). Inflammation and steatosis or new bone formation might exist in two independent processes. After fatty deposition, new bone formation might still progress even if the inflammation subsides (Poddubnyy and Sieper, 2017), and to date, several genetic polymorphism markers are associated with susceptibility to AS diseases. For example, a higher gene dickkopf-1 level blunted Wnt signaling and suppressed new bone formation (Heiland et al., 2012). Single nucleotide polymorphisms rs8092336 were observed to be associated with mSASSS (Cortes et al., 2015), and protein sclerostin was linked with structural damage (Appel et al., 2009). However, the current inflammation-driven scoring system and well-defined markers only reflected the current trends in the disease status and lacked a prognosis score of future outcomes for predicting structural damage. Moreover, studies on the role of genetic polymorphisms in imaging the changes associated with AS were still insufficient. Thus, in-depth exploration of the pathogenesis and a prognostic model for bone bridge formation of AS patients is urgently needed in the era of precision medicine.

Over the past decade, the discovery and characterization of lncRNA revealed diverse regulatory roles, including critical contributions throughout the entire osteogenic differentiation regulatory cascade (Gong et al., 2018). In addition, the lncRNA–miRNA–mRNA interaction could help find the new regulatory mechanism and enrich novel therapeutic strategies (Ju et al., 2020). Based on the lncRNA expression profiles of peripheral whole blood cells obtained by RNA-seq and clinical data, we discovered 205 DElncRNAs and ultimately found two novel lncRNA 326C3.7 and lncRNA 122K13.12, which were highly expressed (about 3 times in AS patients compared with that in healthy people) and could reliably and effectively identify high-risk patients who might develop severe structural damage.

First, we inferred potential functions of lncRNA 326C3.7 and lncRNA 122K13.12 based on coexpression mRNA analysis described in previous subjects. Functional enrichment analysis demonstrated that the coexpressed results were primarily enriched as the TNF (ID: hsa04668) and mitogen-activated protein kinase (MAPK) signaling pathway (ID: hsa04010), which were the core signaling pathways involved in the pathological process of structural damage (Li et al., 2014; Menegatti et al., 2020). Bioinformatics analysis identified some essential genes, including the suppressor of cytokine signaling 3 (SOCS3), TNF actin-interacting protein 3 (AIP3), endothelin-1 (EDN1), activating transcription factor 4 (ATF4), JUN, and 30 miRNAs. SOCS3 is a significant regulator of interleukin-23 and a signal transducer and an activator of transcription 3 (IL23-STAT3) signaling. A study investigated SOCS3 in peripheral blood cells in AS and found that it correlated with the functional ability of the patients, acute-phase reactants, and serum proinflammatory cytokines (Chen et al., 2016). In the animal model study, IL23 promoted osteoblast differentiation via activation of the STAT3 pathway, disrupted SOCS3 expression significantly, and increased phosphorylation of STAT3. Therefore, SOCS3 is a crucial regulator of osteoblast differentiation in spondyloarthritis (Chen et al., 2018). In another study of 667 AS patients and 667 matched healthy controls, tumor necrosis factor alpha-induced protein 3 (TNFAIP3) conferred a protective effect on AS susceptibility (Yang et al., 2019). TNFAIP3, tumor necrosis factor α-induced protein 3, is a gene that encodes for the ubiquitin-modifying enzyme known as A20. A20 participates in mediating immune and inflammatory responses by suppressing the function of nuclear factor-kappaB (NF-κB) (Wullaert et al., 2011; Sun et al., 2013). EDN1 was one of the gene loci of endothelin-1. High levels of plasma EDN1 have been observed in rheumatoid arthritis (Panoulas et al., 2008). ET-1 was positively correlated with the levels of CRP, ESR, and interleukin-6 (IL-6) in AS patients (Przepiera-Będzak et al., 2016). Another three DEmRNAs were also associated with the development of arthritis (Tsushima et al., 2015). In summary, this tells us that the dysregulation of these critical genes may increase susceptibility to AS development. Additionally, these two lncRNAs may be combined with the five DEmRNAs to influence the progression of AS through the TNF signaling pathway.

Based on the AS-specific ceRNA regulatory network constructed in the present study, only two out of the 30 miRNAs previously identified as particularly important in AS were detected. T Yildirim(Yildirim et al., 2021) et al. evaluated 84 miRNA profiles in 2021, and the expressions in AS patients and healthy controls were compared, and both miR-497-5p and miR-511-5p were significantly upregulated in HLA-B27 + patients (*p* = 0.03063). Therefore, in our study, lncRNA profiles, mRNA profiles, and bioinformatics approaches were used to initially explore lncRNA 326C3.7 and lncRNA 122K13.12 in the potential pathogenesis of AS and explore the potential mechanism of regulation through the ceRNA network. Moreover, these two lncRNAs were significantly correlated with many known AS biomarkers. Understanding these novel RNA cross-talks might provide insights into gene regulatory networks with implications in AS and lay a foundation for further functional research.

Next, we further revealed the association between the relative expression value of lncRNA 326C3.7 and clinical indicators. This lncRNA was significantly correlated with some indicators which reflected the structural damage resulting from AS. Linear regression confirmed that the relative expression of lncRNA 326C3.7 (*β* = 0.473, *t* = 3.560, *p* = 0.001) was positively correlated with mSASSS (*R* ^*2*^ = 0.252, *F* = 7.430, and *p* = 0.002). With a higher relative expression value of lncRNA 326C3.7, the mSASSS was also increased. Logistic regression further confirmed that the capacity of lncRNA 326C3.7 [*OR* = 1.324 (1.003–1.748), *p* = 0.048] to predict bone bridge formation was independent of conventional clinical factors, including X-ray stage, time of delayed diagnosis, mSASSS, and SPARCC. Notably, lncRNA 326C3.7 had a high sensitivity (0.800) and specificity (0.605) when used to predict bone bridge formation [AUC was 0.739 (95% CI = 0.609–0.870 and *p* = 0.003)]. lncRNA 326C3.7 could be classified as the most attractive biomarker in modern AS diagnosis and treatment, which could provide accurate prognoses for the most long-term outcome of AS patients, thus helping clinicians choose the most effective treatment.

As interest in the treat-to-target strategy has grown, several disease activity and structural damage scores have been developed to enhance clinical strategies at the present stage. However, the lack of a prediction method for bony bridge formation might result in undesirable outcomes in AS. Therefore, we constructed lncRNA 326C3.7-based nomograms to quantify the probability of bony bridge formation in AS patients. One of the most significant advantages of this nomogram was its simplicity. With the expression of lncRNA 326C3.7 and commonly available clinical variables, including the time of delayed diagnosis and X-ray stage, clinicians could easily estimate long-term outcomes and make decisions for individual AS patients. As expected, this newly proposed prognostic nomogram performed well in predicting bone bridge formation in AS patients, but it still had several limitations. First, although lncRNA 326C3.7 can predict structural damage, it did not appear to be related to inflammatory markers. The specific mechanism still needed verification using subsequent mechanism experiments. Second, our study design was retrospective. In clinical studies, bone bridge formation always took years or even decades, so we did not have enough follow-up samples to verify our prognostic model further. Thus, prospective clinical studies were needed to validate our findings and determine whether the nomogram was accurate and improved outcomes.

In conclusion, we found two novel lncRNAs (326C3.7 and 122K13.12) that significantly increased in AS patients, especially those with a bone bridge. These gene-based ceRNA networks may participate in the development or progression of AS by regulating the TNF signaling pathway. Importantly, using the combination of lncRNA 326C3.7 with conventional clinical risk factors (delayed time and X-ray stage), we developed a nomogram that could accurately predict bone bridge formation in AS patients. This user-friendly prognostic nomogram facilitated the individualized treat-to-target of AS patients.

## Data Availability

The datasets presented in this study can be found in online repositories. The names of the repository/repositories and accession number(s) can be found below: GEO, GSE181364.
