# Supplementary material for: The Potential Regulatory Mechanism of lncRNA 122K13.12 and lncRNA 326C3.7 in Ankylosing Spondylitis
Source: Front Mol Biosci. 2021 Oct 21;8:745441. doi: 10.3389/fmolb.2021.745441 (PMC8566704; doi:10.3389/fmolb.2021.745441)
Supplement: Supplementary file 1 [file Table1.docx]

**Supplementary_Material 1. Baseline clinical characteristics of 68 AS patients involved in this study**

| Indicators | | AS patients | | *t*/*x*^2^/*Z* | *P* |
| --- | --- | --- | --- | --- | --- |
|  |  | Without bone bridge | With bone bridge |  |  |
| N | | 38 | 30 |  |  |
| Age(years) | | 29.3±1.4 | 35.3±3.0 | 10.312 | < 0.001 |
| Male/female | | 27/11 | 21/9 | 0.574 | 0.681 |
| Expression of lncRNA | ENSG00000254910 | 5.067(2.564-6.775) | 8.924(5.909-10.339) | 2.781 | 0.005 |
|  | ENSG00000278238 | 6.606(3.111-8.491) | 6.485(3.946-8.447) | 1.832 | 0.057 |
| Time of delayed diagnosis (years) | | 2.7±0.4 | 5.3±1.0 | 15.284 | 0.004 |
| VAS | | 3.0(3.0-5.0) | 3.0(1.0-5.0) | 2.920 | 0.121 |
| ESR(mm/h) | | 17.0(9.0-38.0) | 15.0(11.0-44.0) | 1.695 | 0.557 |
| CRP(mg/l) | | 6.8(1.8-21.6) | 18.0(9.7-43.5) | 1.285 | 0.673 |
| ASDAScrp | | 2.20(1.45-3.21) | 2.66(2.35-3.74) | 1.877 | 0.773 |
| mSASSS | | 5.0(2.0-10.0) | 25.0(12.0-32.0) | 4.425 | < 0.001 |
| SPARCC | | 17.0(1.0-24.0) | 4.00(1.0-10.0) | 4.173 | 0.196 |
| X-ray stage | 2 | 18 | 6 | 3.152 | 0.017 |
|  | 3 | 18 | 16 |  |  |
|  | 4 | 2 | 8 |  |  |
| With bone marrow edema | | 29 | 9 | 3.717 | 0.032 |
