# Supplementary material for: The Potential Regulatory Mechanism of lncRNA 122K13.12 and lncRNA 326C3.7 in Ankylosing Spondylitis
Source: Front Mol Biosci. 2021 Oct 21;8:745441. doi: 10.3389/fmolb.2021.745441 (PMC8566704; doi:10.3389/fmolb.2021.745441)
Supplement: Supplementary file 2 [file Table2.DOCX]

**Supplementary_Material 2.** Correlation analysis of candidate lncRNAs and clinical-related indicators

| Indicators | | | Delayed time | VAS | ESR | CRP | ASDAScrp | X-ray stage | mSASSS | SPARCC | Bone bridge formation |
| --- | --- | --- | --- | --- | --- | --- | --- | --- | --- | --- | --- |
| Expression of lncRNA | ENSG00000254910 | *r* | 0.371 | 0.371 | 0.029 | 0.062 | 0.259 | 0.381 | 0.390 | 0.380 | 0.380 |
|  |  | *P value* | 0.005 | 0.005 | 0.832 | 0.658 | 0.063 | 0.004 | 0.003 | 0.003 | 0.003 |
|  | ENSG00000278238 | *r* | -0.213 | -0.213 | -0.052 | -0.054 | -0.218 | 0.272 | -0.244 | -0.229 | -0.229 |
|  |  | *P value* | 0.115 | 0.115 | 0.708 | 0.698 | 0.120 | 0.041 | 0.072 | 0.084 | 0.084 |
