# Supplementary material for: The Potential Regulatory Mechanism of lncRNA 122K13.12 and lncRNA 326C3.7 in Ankylosing Spondylitis
Source: Front Mol Biosci. 2021 Oct 21;8:745441. doi: 10.3389/fmolb.2021.745441 (PMC8566704; doi:10.3389/fmolb.2021.745441)
Supplement: Supplementary file 3 [file Table5.DOCX]

**Supplementary_Material 5***.* The primer information

| **gene** | **sequence** |
| --- | --- |
| 245D16.4 | FORWARD: CGTAAACACCGATCTGGCGTACC |
|  | REVERSE: CGTCCCACCTCAGAATTGATGCTC |
|  |  |
| 326C3.7 | FORWARD: GACAGCCTGGATTGCCGACAG |
|  | REVERSE: CCCTGGGAGGAGTTCCTGTGTC |
|  |  |
| β-actin | FORWARD: CTCCATCCTGGCCTCGCTGT |
|  | REVERSE: GCTGTCACCTTCACCGTTCC |
