# Supplementary material for: The Potential Regulatory Mechanism of lncRNA 122K13.12 and lncRNA 326C3.7 in Ankylosing Spondylitis
Source: Front Mol Biosci. 2021 Oct 21;8:745441. doi: 10.3389/fmolb.2021.745441 (PMC8566704; doi:10.3389/fmolb.2021.745441)
Supplement: Supplementary file 4 [file Table3.DOCX]

| Indicators | *β* | 95%CI | *t* | *P_1_* | *R^2^* | *F* | *P_2_* |
| --- | --- | --- | --- | --- | --- | --- | --- |
| ENSG00000254910 | 0.473 | (0.750-2.706) | 3.560 | 0.001 | 0.252 | 7.430 | 0.002 |
| CRP | 0.006 | (-0.117-0.184) | 0.071 | 0.944 |  |  |  |
| ESR | 0.030 | (-0.172-0.167) | 0.304 | 0.763 |  |  |  |
| Delayed time | 0.601 | (-2.217-6.393) | 0.979 | 0.194 |  |  |  |
| ASDAScrp | 0.287 | (0.163-4.813) | 2.157 | 0.037 |  |  |  |

**Supplementary_Material 3.** Linear regression between mSASSS and clinical-related indicators
