# Supplementary material for: The Potential Regulatory Mechanism of lncRNA 122K13.12 and lncRNA 326C3.7 in Ankylosing Spondylitis
Source: Front Mol Biosci. 2021 Oct 21;8:745441. doi: 10.3389/fmolb.2021.745441 (PMC8566704; doi:10.3389/fmolb.2021.745441)
Supplement: Supplementary file 5 [file Table4.DOCX]

| Indicators | Univariate analysis | | Multivariate analysis | |
| --- | --- | --- | --- | --- |
|  | OR (95% CI) | *P* value | OR (95% CI) | *P* value |
| ENSG00000254910 | 1.402(1.110-1.772) | 0.005 | 1.324(1.003-1.748) | 0.048 |
| Delayed time | 1.315(1.007-1.607) | 0.007 | 1.289(1.018-1.632) | 0.035 |
| X-ray stage | 7.627(2.225-26.149) | 0.001 | 4.613(1.202-17.696) | 0.026 |

**Supplementary_Material 4.** Univariate and multivariate logistic regression analysis of bone bridge formation and indicators
