# Supplementary material for: The Potential Regulatory Mechanism of lncRNA 122K13.12 and lncRNA 326C3.7 in Ankylosing Spondylitis
Source: Front Mol Biosci. 2021 Oct 21;8:745441. doi: 10.3389/fmolb.2021.745441 (PMC8566704; doi:10.3389/fmolb.2021.745441)
Supplement: Supplementary file 6 [file Table6.DOCX]

Supplementary Material 6. Functional enrichment analysis of DElncRNAs

| **GO term** | **Description** |
| --- | --- |
| GO:0033687 | osteoblast proliferation |
| GO:0001649 | osteoblast differentiation |
| GO:0071356 | cellular response to tumor necrosis factor |
| GO:0032720 | negative regulation of tumor necrosis factor production |
| GO:0032691 | negative regulation of interleukin-1 beta production |
| GO:0071347 | cellular response to interleukin-1 |
| GO:0032743 | positive regulation of interleukin-2 production |
| GO:0034136 | negative regulation of toll-like receptor 2 signaling pathway |
| GO:0034140 | negative regulation of toll-like receptor 3 signaling pathway |
| GO:0032713 | negative regulation of interleukin-4 production |
| GO:0034144 | negative regulation of toll-like receptor 4 signaling pathway |
| GO:0034148 | negative regulation of toll-like receptor 5 signaling pathway |
| GO:0032715 | negative regulation of interleukin-6 production |
| GO:0032757 | positive regulation of interleukin-8 production |
| GO:0032733 | positive regulation of interleukin-10 production |
| GO:0032735 | positive regulation of interleukin-12 production |
| GO:0032736 | positive regulation of interleukin-13 production |
| GO:0045598 | regulation of fat cell differentiation |
| GO:0009612 | response to mechanical stimulus |
| GO:0045600 | positive regulation of fat cell differentiation |
| GO:0034124 | regulation of MyD88-dependent toll-like receptor signaling pathway |
| GO:0043374 | CD8-positive, alpha-beta T cell differentiation |
| GO:0045084 | positive regulation of interleukin-12 biosynthetic process |
| GO:0045590 | negative regulation of regulatory T cell differentiation |
| GO:0071260 | cellular response to mechanical stimulus |
| GO:0006954 | inflammatory response |
| GO:0050776 | regulation of immune response |
| GO:0051023 | regulation of immunoglobulin secretion |
| GO:0042100 | B cell proliferation |
| GO:0042511 | positive regulation of tyrosine phosphorylation of Stat1 protein |
| GO:0045600 | positive regulation of fat cell differentiation |
| GO:0045684 | positive regulation of epidermis development |
| GO:0045892 | negative regulation of transcription, DNA-dependent |
| GO:0003723 | RNA binding |
| GO:0008270 | zinc ion binding |
| GO:0003677 | DNA binding |
| GO:0005515 | protein binding |
| GO:0006355 | regulation of transcription, DNA-templated |
| GO:0005737 | cytoplasm |
| GO:0005634 | nucleus |

| **KEGG ID** | **Description** |
| --- | --- |
| hsa04668 | TNF signaling pathway |
| hsa04380 | Osteoclast differentiation |
| hsa04657 | IL-17 signaling pathway |
| hsa03320 | PPAR signaling pathway |
| hsa04010 | MAPK signaling pathway |
| hsa04064 | NF-kappa B signaling pathway |
| hsa04620 | Toll-like receptor signaling pathway |
| hsa04670 | Leukocyte transendothelial migration |
| hsa04310 | Wnt signaling pathway |
| hsa05321 | Inflammatory bowel disease |
